# Update on GM canola crops as novel sources of omega‐3 fish oils

**DOI:** 10.1111/pbi.13045

**Published:** 2018-12-13

**Authors:** Johnathan A. Napier, Rolf‐Erik Olsen, Douglas R. Tocher

**Affiliations:** ^1^ Department of Plant Sciences Rothamsted Research Harpenden UK; ^2^ Department of Biology Norwegian University of Science and Technology Trondheim Norway; ^3^ Faculty of Natural Sciences Institute of Aquaculture University of Stirling Stirling UK

**Keywords:** Canola, omega‐3 fish oils, regulatory approval, APHIS

There is considerable interest in new sources of omega‐3 long‐chain (here defined as fatty acids ≥C20) polyunsaturated fatty acids (LC‐PUFA), specifically eicosapentaenoic acid (EPA; 20:5n‐3) and docosahexaenoic acid (DHA; 22:6n‐3), commonly known as omega‐3 fish oils, to supplement the limited supplies of oceanic fish oil (Tocher, 2015). These alternative sources include extraction of other diverse marine organisms (e.g. krill, plankton etc.), algal fermentation and the genetic engineering of microbes such as yeasts. Another approach is the synthesis of omega‐3 fish oils in transgenic plants (reviewed in Napier *et al*., 2015), and this short article will discuss the recent results obtained by two major industry collaborations.

The objective of engineering plants to accumulate EPA and DHA has long been recognized as worthy (summarized in Domergue *et al*., 2005). Genes for this biosynthetic pathway (Figure 1) were characterized by the early 2000s, but efficient transgenic reconstitution proved more challenging. Varying levels of EPA and DHA have been reported in both Arabidopsis and Camelina by different groups (comprehensively discussed in Napier *et al*., 2015). However, significant commercial effort has been also focussed on canola (*Brassica napus*) as the host for this transgenic pathway, since genetic and agronomic resources are well‐established for this crop, with also significant grower acceptance of GM canola in N. America.

**Figure 1:**
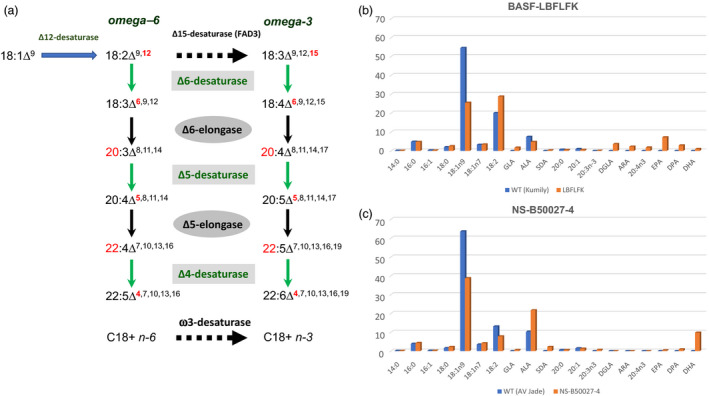
*The biosynthetic pathway for LC‐PUFAs and efficacy in transgenic canola*. (a) Schematic representation of the enzymatic biosynthesis of LC‐PUFAs. The two different ‘tracks’ (omega‐6, omega‐3) are shown, as are the individual enzyme activities required for each step; this modification is shown in red. (b) Total seed fatty acid composition for canola event LBFLFK, compared with the parent line *Kumily*. Data are aggregated results from multi‐location field trials [Sottosanta *et al*. (2018) Table 32,33]. (c) Total seed fatty acid composition for canola event NS‐B50027‐4, compared with the parent line *AV‐Jade*. Data are aggregated results from multi‐location field trials [Devine *et al*., 2017; table 15].

Until very recently, the only substantial data on producing omega‐3 LC‐PUFAs in transgenic canola were limited to one study (Walsh *et al*., 2016) from Dow & DSM, where genes for the anaerobic PKS pathway from *Schizochytrium* were expressed in a seed‐specific manner in canola. This resulted in modest levels (<4%) of DHA, though the stability of the trait in the field was demonstrated. However, two separate industry co‐operations (BASF & Cargill; Nuseed, CSIRO & GRDC) have also been developing GM canola engineered with the aerobic (desaturase and elongase) pathway (Figure 1a) previously studied in *Arabidopsis* and *Camelina* (reviewed in Napier *et al*., 2015). The recent publications in the USA of the USDA Animal and Plant Health Inspection Service (APHIS) ‘Petition for non‐regulatory status’ (Connelly and MacIntosh, 2018; Sottosanta *et al*., 2018) by these two different teams provide an insight into progress in some of the most advanced metabolic engineering attempted in transgenic plants, and will be discussed individually below. It should be noted that approval by APHIS allows for agricultural cultivation, but additional approvals from the US Food and Drug Administration (FDA) are required for feed and food use. It is not our aim to rank one oil over the other, since both represent significant scientific advances in plant metabolic engineering. Rather, we summarize the different molecular approaches to generating omega‐3 LC‐PUFAs in transgenic canola.

The dossier for regulatory approval of the unique canola event ‘LBFLFK’ submitted by BASF provides significant data on the various criteria which are used by APHIS to determine regulatory status (Sottosanta *et al*., 2018). This includes molecular details of the construct used to generate the omega‐3 LC‐PUFA trait, and also the fatty acid composition of the seed oil. The genetic background for transgenesis was the variety ‘*Kumily*’, which is devoid of erucic acid but has significant amounts of oleic acid in the seed oil. The transgene cassette contains all seven of the activities listed in the pathway shown in Figure 1a, specifically, a Δ12‐desaturase, Δ6‐desaturase, Δ6‐elongase, Δ5‐desaturase, Δ5‐elongase, Δ4‐desaturase and ω3‐desaturase. Each gene encoding an activity is under the regulation of a seed‐specific promoter. In event LBFLFK, several activities are represented more than once – two copies of the Δ6‐elongase and the Δ5‐desaturase, and three copies of the ω3‐desaturase. In these cases, genes from different organisms are used – for example, (synthetic) genes encoding the Δ6‐elongase activity from both *Physcomitrella patens* and *Thalassiosira pseudonana* were used. Therefore, the transgene cassette contains 12 seed‐specifically expressed omega‐3 LC‐PUFA biosynthetic genes (plus the AHAS gene which provides resistance against imadazolinone herbicides), representing a large transgene insert of ~ 44Kb. Whole genome resequencing of the LBFLFK event revealed that this cassette was present twice in this transgenic canola line, being present intact on chromosomes C03 and Cnn. Thus, in total, 24 transgenes for the biosynthesis of EPA and DHA are present in event LBFLFK. In terms of the seed oil fatty acid profile, the aggregated data from two years multi‐location field trials (Sottosanta *et al*., 2018) indicate that the LBFLFK event in the *Kumily* background produces EPA in preference to DHA (Figure 1b).

As detailed in the Nuseed petition to APHIS (Connelly and MacIntosh, 2018), the transgenic canola event NS‐B50027‐4 has been selected for deregulation. The genetic background used for transformation is the variety ‘*AV Jade*’, which is also a zero‐erucic acid type, but has higher levels of oleic acid than the *Kumily* variety used by BASF. The transgene cassette used to introduce the omega‐3 LC‐PUFA trait contains genes encoding all of the activities shown in Figure 1a, with each gene being under the regulation of a seed‐specific promoter. Unlike BASF's LBFLFK event, each biosynthetic activity is represented by a single gene, and herbicide tolerance to glufosinate is conferred by the phosphinothricin acetyltransferase gene, meaning the predicted overall size of the insertion is ~23 Kb. Based on genomic resequencing, in B50027‐4 there are two unlinked insertion sites – a duplicate, head‐to‐tail insertion of the cassette at chromosome A05 (~46 Kb integration) and a partial insertion of ~12 Kb at A02. In the latter case, only four genes (Δ6‐desturase, Δ5‐desaturase, Δ5‐elongase, ω3‐desaturase) were integrated. Thus, in total 18 transgenes for the biosynthesis of EPA and DHA are present in event NS‐B50027‐4. In event NS‐B50027‐4, the source organisms from which the biosynthetic activities were obtained are different from those described for the BASF LBFLFK event. In terms of the seed oil fatty acid profile, the aggregated data from two years multi‐location field trials (Connelly and MacIntosh, 2018; Devine *et al*., 2017) indicate that the NS‐B50027‐4 event in the *AV Jade* background produces DHA in preference to EPA, as shown in Figure 1c. Recently, APHIS have affirmed the non‐regulated status of NS‐B50027‐4.

Both the BASF and Nuseed canola events generated higher total levels (~12%) of omega‐3 LC‐PUFAs than previously reported (Walsh *et al*., 2016) confirming the superiority of the aerobic pathway, despite it requiring a greater number of individual enzyme activities. Perhaps more surprising is the different seed oil profiles present in LBFLFK and NS‐B50027‐4. Specifically, LBFLFK produces a seed oil which contains ~7% EPA, ~3%DPA and ~1%DHA, whereas NS‐B50027‐4 has an oil containing <0.5% EPA, ~1%DPA and ~10% DHA (Figure 1a–c). In addition, compared to the host varieties into which the transgenes were introduced, LBFLFK has an increased level of 18:2n‐6 and decreased 18:3n‐3, whereas NS‐B50027‐4 has increased proportions of both 18:2n‐6 and 18:3n‐3. In terms of the increased accumulation of 18:2n‐6 in LBFLFK, this may be due to the activity of the *Phytophthora soja* Δ12‐desaturase, responsible for the conversion of oleic acid to 18:2n‐6. Interestingly, the NS‐B50027‐4 event also contains a Δ12‐desaturase, from the yeast *Lachancea kluyveri* and, whilst this also generates 18:2n‐6, the presence of a broad‐specificity (C_18+_) ω3‐desaturase from *Pichia pastoris* likely helps to promote (in conjunction with the endogenous FAD3 gene) conversion of this to 18:3n‐3. In turn, the latter is the preferred substrate of the *Micromonas pusilla* Δ6‐desaturase, skewing the pathway towards the omega‐3 track, whereas the *Ostreococcus tauri* Δ6‐desaturase present in LBFLFK prefers omega‐6 substrates (Napier *et al*., 2015; Petrie *et al*., 2010). Similarly, the very high efficiency of the *Pyramimonas cordata* Δ5‐elongase present in NS‐B50027‐4 likely favours the accumulation of DHA over EPA (Petrie *et al*., 2010; discussed in Napier *et al*., 2015). Collectively, this may explain some of the differences between LBFLFK and NS‐B50027‐4.

The agricultural validation of transgenic canola plants accumulating of omega‐3 LC‐PUFAs in their seed oils confirms the original promise on which the concept of a land‐based source of fish oils was founded. With regulatory approval, two new commodity sources of plant‐derived omega‐3 fish oils will soon be available. But how might such oils be used? Given that around 75% of marine‐sourced fish oils are currently used in aquaculture, with almost 80% of that going to feeds for salmonids and marine fish (IFFO, 2017), this seems the most likely option. Indeed, the finite and limiting supply of traditional marine ingredients such as fish oils and meals, until now the only major source of EPA and DHA for fish diets, has been insufficient to support the growth of aquaculture resulting in existing supplies being spread thinner and thinner in the increasing volume of commercial feeds, resulting in significantly reduced levels of EPA and DHA in the resulting farmed fish (Sprague *et al*., 2016). Therefore, application of the new agricultural crop‐based sources of EPA and DHA in aquafeeds has the potential to restore and/or enhance levels in farmed fish, maintaining or improving their nutritional quality for consumers. As fish and seafood have always been the way we have obtained EPA and DHA in our diet and, after much effort to inform the public on omega‐3 and their food sources, this is now well appreciated by consumers, aquaculture seems the logical approach. However, given that terrestrial agriculture offers the possibility of a new and abundant supply of omega‐3 LC‐PUFA, this could open up the possibility to fortify other foods such as poultry, pork and eggs, which although less efficient than fish, can provide these key nutrients to the significant number of people that do not consume fish and seafood. Furthermore, direct human consumption via capsules, currently the other main use for marine fish oil accounting for almost 20% of supply, may also be feasible. Of course, a number of practical issues remain as to how these new GM crops will be grown and processed, but this will likely involve strong agricultural stewardship and identity preservation to ensure a closed production system for this trait. It may also be the case that different genetic backgrounds (varieties) can further enhance the accumulation of EPA and DHA in canola.

## Conclusions

Two new plant‐based sources of omega‐3 LC‐PUFA are expected to soon have full regulatory approval in the US, presenting a sustainable, terrestrial source of omega‐3 fish oils. Given the importance of omega‐3 LC‐PUFA in maintaining optimal health in livestock, farmed fish and in human consumers, *de novo* sources of these fatty acids represent both a wide‐reaching advance and a validation of the ability of plant biotechnology to deliver benefit to the consumer.
